# Neurotrophic actions of dopamine on the development of a serotonergic feeding circuit in *Drosophila melanogaster*

**DOI:** 10.1186/1471-2202-13-26

**Published:** 2012-03-13

**Authors:** Wendi S Neckameyer, Parag Bhatt

**Affiliations:** 1 Department of Pharmacological and Physiological Science, Saint Louis University School of Medicine, 1402 South Grand Boulevard, Saint Louis, Missouri 63104, USA

## Abstract

**Background:**

In the fruit fly, *Drosophila melanogaster*, serotonin functions both as a neurotransmitter to regulate larval feeding, and in the development of the stomatogastric feeding circuit. There is an inverse relationship between neuronal serotonin levels during late embryogenesis and the complexity of the serotonergic fibers projecting from the larval brain to the foregut, which correlate with perturbations in feeding, the functional output of the circuit. Dopamine does not modulate larval feeding, and dopaminergic fibers do not innervate the larval foregut. Since dopamine can function in central nervous system development, separate from its role as a neurotransmitter, the role of neuronal dopamine was assessed on the development, and mature function, of the 5-HT larval feeding circuit.

**Results:**

Both decreased and increased neuronal dopamine levels in late embryogenesis during development of this circuit result in depressed levels of larval feeding. Perturbations in neuronal dopamine during this developmental period also result in greater branch complexity of the serotonergic fibers innervating the gut, as well as increased size and number of the serotonin-containing vesicles along the neurite length. This neurotrophic action for dopamine is modulated by the D_2 _dopamine receptor expressed during late embryogenesis in central 5-HT neurons. Animals carrying transgenic RNAi constructs to knock down both dopamine and serotonin synthesis in the central nervous system display normal feeding and fiber architecture. However, disparate levels of neuronal dopamine and serotonin during development of the circuit result in abnormal gut fiber architecture and feeding behavior.

**Conclusions:**

These results suggest that dopamine can exert a direct trophic influence on the development of a specific neural circuit, and that dopamine and serotonin may interact with each other to generate the neural architecture necessary for normal function of the circuit.

## Background

Classical neurotransmitters can function as growth factors in developing neural tissues before adopting their roles as signaling molecules in the mature nervous system, and thus can exert pleiotropic effects on nervous tissue function [1,2]. Impaired neuronal signaling arising from developmental perturbations has been strongly implicated in the etiology of depression [3,4], and autism spectrum disorders [5]. Specifically, deficiencies in developmental dopamine (DA) signaling contribute to numerous psychiatric symptoms [6], and DA dysfunction plays a critical role in the onset of schizophrenia [7]. Developmental abnormalities in serotonin (5-HT) pathways have also been implicated in schizophrenia [8] as well as in autism [9,10].

Elegant studies by Lauder and Kater and colleagues demonstrated that altered DA or 5-HT levels during embryogenesis affected the synaptic architecture of mature neurons [11-15]. Later studies confirmed that 5-HT is critical for development of synaptic connectivity [16]. Using a simple model to assess both development and function of a specific behavioral circuit in the larval stage of the fruit fly (*Drosophila melanogaster*), we demonstrated an inverse relationship between developmental 5-HT levels and the complexity of 5-HT-immunoreactive fibers projecting from the brain to the foregut. These also correlated with perturbations in larval feeding, the functional output of the circuit [17]. This developmental role for 5-HT was distinct from its actions as a neurotransmitter.

Although DA is also thought to play a neurotrophic role in central nervous system (CNS) development, these studies have been largely limited to cell culture systems, and have focused on the role of different DA receptors. DA has been shown to cause a transient retraction in the growth cone fillipodia in specific neurons in the pond snail [18]. Similarly, DA, acting through the D_1 _receptor, inhibited growth cone motility in cultured retina neurons [19]. D_2 _receptors have been shown to affect the number and extent of neurite branching in cultured neuronal cell lines, as do D_3 _and D_4 _receptors [20,21].

**Figure 1:**
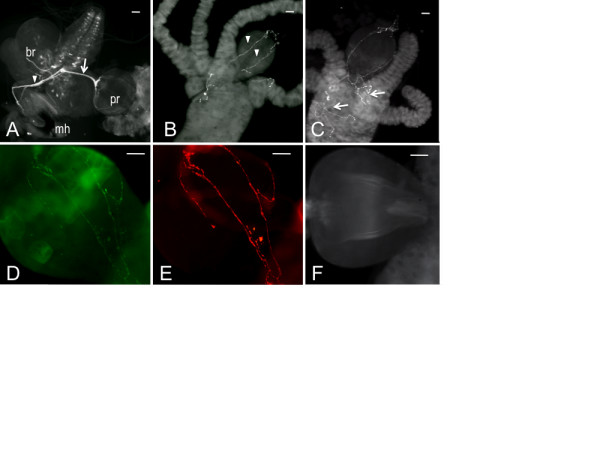
**The *Drosophila *larval feeding circuit is not innervated by dopaminergic fibers**. Gut tissues dissected from *Drosophila *3^rd ^instar larvae were immunostained with an antibody raised against *Drosophila *neuronal tryptophan hydroxylase (DTRH, A) or 5-HT (B, C). A. mh, mouth hooks; pr, proventriculus; br, brain (showing the pattern of 5-HT neurons). The arrowhead designates the frontal nerve and an arrow the recurrens nerve. B. proventriculus showing axonal fibers (denoted with arrowheads). C. fibers fasiculating in the midgut (denoted with arrows). D - E. proventricular fibers from larvae carrying a neuronally expressed green fluorescent protein tagged to synaptotagmin. E, GFP fluorescence; D, anti-DTRH. F. Gut tissues stained with an antibody raised against *Drosophila *tyrosine hydroxylase (DTH). Scale bars = 20 μm.

There are less than 100 DA neurons in the larval *Drosophila *CNS. The stereotypical DA pattern is first detected at stage 15 (18-20 hours after egg laying, which is approximately 3 hr before hatching), and is fully established before the end of embryogenesis. All DA neurons appear to be interneurons [22,23]. The approximately 100 larval 5-HT neurons are bilaterally symmetric and are spatially distinct from the DA neurons [24]. The larval 5-HT neurons innervate the pharyngeal muscles, the proventriculus, and the midgut, consistent with a role in larval feeding (see Figure 1). 5-HT cell bodies and some projections can be visualized in the CNS by 16-18 hr after egg laying; no peripheral fibers are present until two hours later, and the 5-HT fibers innervating the gut are not observed until 20-22 hr [25].

Some studies have suggested that both DA and 5-HT affect neuroblast proliferation, neurite branching, and/or neurite extension, but the mechanisms for these effects have not been fully elucidated, and it is not known whether DA and 5-HT compete with each other during the process of neurite extension and targeting, and thus compete for functional interactions. Neonatal lesioning of 5-HT projections to the rodent medial prefrontal cortex is associated with an increase in adult TH-immunoreactive varicosities [26]. However, application of 5-HTP (the direct precursor of 5-HT), but not DA, decreased the number of branch points of a defined 5-HT neuron in *Helisoma *[27]. More critically, few studies have addressed the behavioral consequences of the effects of DA or 5-HT developmental dysfunction on a mature neural circuit. To begin to elucidate the mechanisms by which DA and 5-HT regulate CNS development, we assessed the role of neuronal DA during late embryogenesis on the development, and mature function, of the 5-HT larval feeding circuit.

## Results

### The *Drosophila *larval feeding circuit

The general structure of the larval stomatogastric nervous system is highly conserved across insect species [28]. The 5-HT feeding circuit is an integral part of the stomatogastric pattern generator, consistent with the fact that 5-HT modulates appetitive behavior in all species including *Drosophila *[17]. Unlike adults, *Drosophila *larvae feed continuously if placed in an appropriate food source [29], in anticipation of the enormous energy demands for metamorphosis. The larval mouth hooks (forming the most anterior part of the cephalopharyngeal plates) shovel food into the gut at a relatively constant rate. Bundled 5-HT nerve fibers connect neurons within the subesophageal ganglion to the cephalopharyngeal plates via the frontal nerve (denoted by the arrowhead in Figure 1A) as well as to the proventriculus (foregut) via the recurrens nerve (denoted by arrow, Figure 1A). The frontal nerve is responsible for the generation of feeding-related motor patterns [28]. The recurrens nerve fasciculates in the proventriculus into individual axonal fibers containing 5-HT-containing vesicles (denoted by arrowheads in Figure 1B); additional fibers branch off when they reach the midgut (denoted by arrows in Figure 1C). Synaptotagmin fused to green fluorescent protein, under the control of the pan-neuronal *elav *promoter, demonstrates 1:1 colocalization with *Drosophila *neuronal tryptophan hydroxylase (DTRH, the rate-limiting step in 5-HT synthesis) within the fibers, evidence that the fibers are axons projecting from central presynaptic neurons (DTRH is responsible for synthesis of neuronal 5-HT [30]) (Figure 1D, E). No tyrosine hydroxylase-immunoreactive fibers innervate the gut (Figure 1F), suggesting that, unlike 5-HT, DA neurotransmission does not modulate larval feeding. This is consistent with the observation that larvae systemically depleted of DA as 2^nd ^to 3^rd ^instars display normal feeding behavior [31].

### Reduced TH levels in the embryonic CNS affect development of the 5-HT feeding circuit

**Figure 2:**
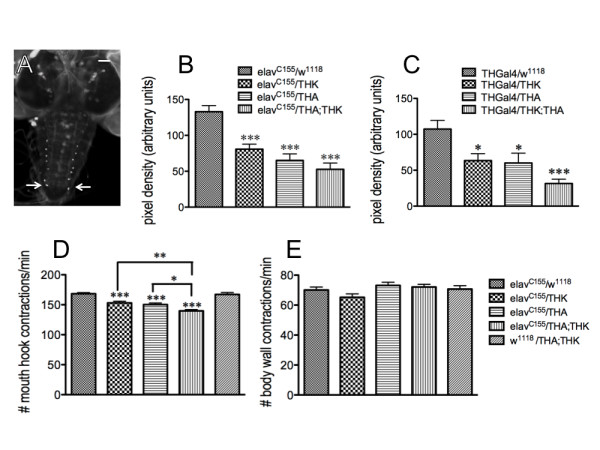
**Constitutive reduction in neuronal DTH results in depressed feeding behavior**. Two independent RNAi transgenic lines (DTHA, inserted on chromosome 2, and DTHK, on chromosome 3) were used to titrate constitutive knockdown of DTH levels throughout CNS development. The pixel intensity of the DTH-immunofluorescence of the most distal dorsolateral neurons in the ventral ganglion was determined to ascertain the extent of DTH knockdown (**A**). Scale bar = 50 μm. Larval brains were dissected from each genotype using the pan-neuronal *elav^C155 ^*(**B**) or THGal4 drivers (**C**). Brains from all animals were assessed in parallel. The DA cell pattern in each brain was visualized with a polyclonal antibody raised against DTH (Neckameyer et al, 2000) and viewed under fluorescence. *elav/w^1118^*, n = 30 neurons [control]; *elav*/THK, n = 18 neurons; *elav*/THA, n = 15 neurons; *elav*/THA;THK, n = 13 neurons; THGal4/*w^1118^*, n = 30 neurons [control]; THGal4/THK, n = 22 neurons; THGal4/THA, n = 18 neurons; THGal4/THA;THK, n = 26 neurons. *p < 0.05, ***p < 0.001, one way ANOVA followed by Tukey's post-test. The animals were then assayed for feeding (**D**) and locomotor behavior (**E**) (including an additional control, *w^1118^*/THA;THK). E. Locomotion was unaffected. *p < 0.05, **p < 0.01, ***p < 0.001, one way ANOVA followed by Tukey's multiple comparisons post-test. n = 40 for behavioral analyses, from 4-6 independent experiments. Lines above the graph depict standard error of the mean.

To reduce levels of neuronal DA, we generated transgenic RNAi lines to knockdown levels of *Drosophila *tyrosine hydroxylase (DTH), which is the first and rate-limiting enzyme in DA synthesis. We identified two transgenic lines (THA, on chromosome 2, and THK, on chromosome 3) and manipulated them singly, and in combination, to effect a titration of DTH knockdown in the CNS. Since expression of DTH is limited to less than 100 neurons in the larval CNS, demonstration of reduced neuronal DTH could not be assessed by changes in whole body DTH enzyme activity or protein levels. Quantitating DA levels strictly within the CNS would also be difficult, since DA is found in the circulating hemolymph as well as in the fat body and ring gland, which are in close proximity to the larval brain. Therefore, the intensity of DTH immunofluorescence of the two most distal dorsolateral neurons in the ventral ganglion (denoted by arrows in Figure 2A) was compared between control animals (*w^1118^*, the parental line for generation of the transgenics), animals carrying one copy of the transgene (THA or THK), and animals carrying both THA and THK. These neurons were chosen because they were distinct and easily identifiable, and essentially free of the TH-immunoreactive neuropil within the ventral nerve cord. To confirm extent of the knockdown, two independent drivers were used: *elav^C155^*, which drives expression throughout the CNS (Figure 2B), and THGal4, which drives expression in the majority of DA neurons, including the distal dorsolateral pair, as well as in other, non-neuronal tissues (Figure 2C). Brains were dissected from each genotype and assessed in parallel under identical conditions. Each neuron was photographed at the same exposure and magnification, and the relative fluorescence of each was determined by quantitating the pixel intensity of a circular sampling region placed at seven different locations within the cytoplasm of each cell. This number was averaged to give a score for each neuron. Under the control of either driver, both THA and THK are capable of reducing DTH levels to a roughly similar extent; the combination of THA and THK reduces expression even further, demonstrating that the degree of knockdown can be titrated. The decrease in DTH levels correlates with reduction in larval feeding in animals where TH has been constitutively reduced in the CNS during development of the circuit (Figure 2D); all feeding assays were accomplished using late 2^nd ^- early 3^rd ^instar larvae. Note that *elav*/DTHA results in reduced feeding relative to *elav*/DTHK animals, consistent with the stronger knockdown of DTHA using the *elav *driver (Figure 2B). To demonstrate that the genetic manipulations did not affect the larva's ability to extend and retract the mouth hooks, body wall contractions for each animal were measured. On an agar surface, the larvae extend and then retract their mouth hooks into the agar to initiate the body wall contractions; the imprint from their mouth hooks is left in the agar substrate. Since this behavior was unaffected (Figure 2E), the defect in feeding cannot be due to motor deficits or other generalized perturbations in physiology.

**Figure 3:**
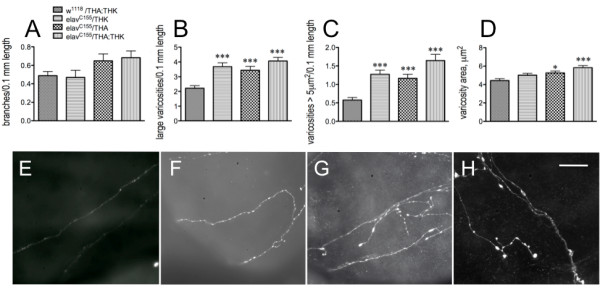
**Constitutive reduction in neuronal DTH results in results in aberrant gut fiber architecture**. Analysis of proventricular tissues from 3^rd ^larval instars dissected and incubated with anti-5-HT. A. Neurite branching. B. Number of large varicosities (> 1 μm^2^) per 0.1 mm neurite length. C. Number of varicosities > 5 μm^2 ^per 0.1 mm length. D. varicosity area. *p < 0.05, ***p < 0.001, one way ANOVA followed by Dunnett's multiple comparisons post-test. *w^1118^*/THA;THK, n = 66 fibers from 48 guts from 8 independent experiments; *elav*/THK, n = 40 fibers from 18 guts from 2 independent experiments; *elav*/THA, n = 37 fibers from 21 guts from 2 independent experiments; *elav*/THA;THK, n = 35 fibers from 18 guts from 3 independent experiments. Lines above the graph depict standard error of the mean. E - H. Visual depiction of 5-HT axonal fibers from *w^1118^*/THA;THK (**E**), *elav*/THK (**F**), *elav*/THA (**G**) and *elav*/THA;THK (**H**) guts. Scale bar = 30 μm.

Reduced neuronal 5-HT during development of this circuit results in increased complexity of the architecture of the 5-HT axonal fibers projecting to the proventriculus: these fibers displayed increased branching, greater numbers of 5-HT-containing vesicles, and significantly increased vesicle size [17]. To determine whether the reduction in neuronal DA similarly correlates reductions in feeding behavior with increased complexity of the 5-HT axonal projections, these parameters were assessed in control and TH knockdown animals (Figure 3). All immunohistochemical analyses were performed using late 3^rd ^instar larvae. While not significant, there is a trend towards increased branching of the 5-HT axonal fibers (Figure 3A). However, the number (Figure 3B) and size (Figure 3C) of the 5-HT-containing vesicles are significantly increased with increasing knockdown of DTH; overall, the reduction in DTH levels (and therefore DA synthesis) correlates with increased vesicle area (Figure 3D). Varicosity number is also increased (data not shown). This can be visually observed by the increasing complexity of the fiber appearance when comparing *w^1118^*/THA;THK (Figure 3E) with *elav*/THK (Figure 3F), *elav*/THA (Figure 3G) and *elav*/THA;THK (Figure 3H) guts. Thus, increasing reductions in DTH levels directly correlate with reductions in feeding (Figure 2), as well as with increases in the number and size of the 5-HT-containing vesicles. These results demonstrate that neuronal DA affects development, and thus mature function, of the 5-HT feeding circuit.

**Figure 4:**
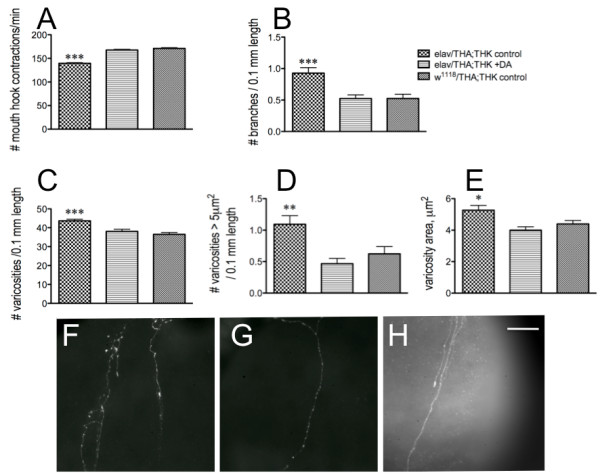
**Exposure of *elav*/THA;THK late-stage embryos to exogenous DA rescues both the feeding and axonal fiber defects**. A. Feeding. *elav*/THA;THK 16 hr old embryos were dechorionated, devitinellated, and placed in serum-free media until hatching (control), or in serum-free media plus 10^-6 ^M DA-HCl (+DA), and compared with *w^1118^*/THA;THK 16 hour old embryos placed in serum-free media until hatching. n = 40 for behavioral analyses, from 4-6 independent experiments. B-E. Analysis of the 5-HT axonal fibers projecting to the foregut. B. Number of branches per 0.1 mm length along the axonal fiber. C. Number of total varicosities per 0.1 mm length; D. Number of varicosities > 5 μm2 per 0.1 mm length; E. Varicosity area. *w^1118^*/THA;THK, 38 fibers from 30 guts from 8 independent experiments; *elav*/THA;THK -DA, 40 fibers from 26 guts from 9 independent experiments; *elav*/THA;THK + DA, 43 fibers from 29 guts from 10 independent experiments. *p < 0.05, **p < 0.01, ***p < 0.001. Statistical analyses were performed using one way ANOVAS and Dunnet's post-tests. Lines above the graph depict standard error of the mean. F - H. Visual depiction of 5-HT axonal fibers from *elav*/THA;THK (**F**), *elav*/THA;THK + DA (**G**), and *w^1118^*/THA;THK (**G**) guts. Scale bar = 30 μm.

To conclusively demonstrate that this was a developmental effect, as opposed to the actions of DA as a neurotransmitter, 16-hour old *elav*/THA;THK embryos (the double transgenic line, providing the greatest reduction in DTH) were exposed for the last 6 hours of embryogenesis either to serum-free media or to serum-free media plus 10^-6 ^M DA-HCl. Once hatched, these animals were removed from the medium, placed on normal food, and assayed for feeding behavior as late 2^nd ^- early 3^rd ^instar larvae. They were compared with *w^1118^*/THA;THK animals exposed only to serum-free media during this stage of embryonic development (control). The exogenous DA rescued the feeding defects in *elav*/THA;THK larvae, resulting in behavior indistinguishable from that of the control (Figure 4A). As before, mouth hook contractions during locomotion were normal (data not shown). We then assessed the gut fiber architecture of these animals. The increase in fiber branching (Figure 4B), total varicosity number (Figure 4C), number of varicosities greater than 5 μm^2 ^(Figure 4D), and varicosity area (Figure 4E) of *elav*/THA;THK axonal fibers projecting to the proventriculus were rescued to control levels by exposure to DA during the last 6 hours of embryogenesis. This can be observed visually when comparing the 5-HT axonal fibers from fibers from *elav*/THA;THK (F), *elav*/THA;THK + DA (G), and *w^1118^*/THA;THK (H) guts. This result conclusively demonstrated that the development of the 5-HT feeding circuit, and thus larval feeding behavior, is sensitive to DA levels during development of the larval feeding circuit.

### Increased TH levels in the embryonic CNS also affect development of the 5-HT feeding circuit

**Figure 5:**
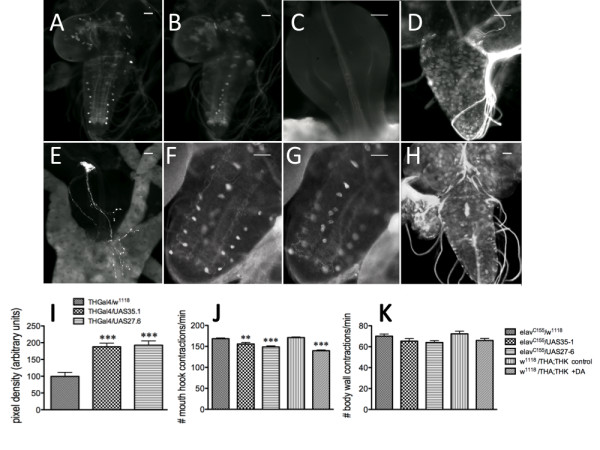
**Constitutive induction in neuronal DTH or increased levels of systemic DA during late embryogenesis results in depressed feeding behavior in larvae**. Two independent UAS transgenic lines (35.1 and 27.6, on chromosomes 2 and 3, respectively) were used to increase neuronal DTH levels. A - C. DTH immunoreactivity in the ventral ganglion of *w^1118^*/UAS27.6 larvae. A. Dorsolateral neurons. B. Medial neurons). C. Proventriculus. D - E. DTH immunoreactivity from *elav^C155^*/UAS27.6 larvae. D, larval brain. E. Proventriculus visualized with anti-DTH. F - H. DTH immunoreactivity in sibling progeny from a UAS35.1/*Bc^1 ^*x *elav^C155 ^*cross (*Bc *[*Black cell*] is a larval marker). F. Dorsolateral DA neurons visualized with anti-DTH in a *Bc^1^*/UAS35.1 larval ventral ganglion. G. Medial DA neurons visualized with anti-DTH in a *Bc^1^*/UAS35.1 3^rd ^instar larval ventral ganglion. H. DTH immunoreactivity in an *elav*/UAS35.1 larval brain. Scale bar = 50 μm. I. Relative pixel intensity of the most distal pair of dorsolateral neurons in THGal4/*w^1118^*, THGal4/UAS35.1 and THGal4/UAS27.6 larvae. Third instar larval brains from the different genotypes were dissected and incubated with anti-DTH antibody. Brains from all animals were assessed in parallel. THGal4/*w^1118^*, n = 12 neurons [control]; THGal4/UAS35.1, n = 24 neurons; THGal4/UAS27.6, n = 15 neurons. ***p < 0.001, one-way ANOVA followed by Tukey's multiple comparisons post-test. The animals were then assayed for feeding (**J**) and compared with 16 hr old control (*w^1118^;*THA;THK) embryos were exposed to serum-free media with or without 10^-6^M DA for 6 hours until hatching. K. Locomotion was unaffected. **p < 0.01, ***p < 0.001, one-way ANOVA followed by Tukey's multiple comparisons post-test. n = 40 for behavioral analyses, from 4-6 independent experiments. Lines above the graph depict standard error of the mean.

To assess the effects of constitutive overexpression of neuronal DTH on the feeding circuit, we generated transgenic lines to induce DTH levels (35.1 on chromosome 2, and 27.6 on chromosome 3). DTH immunofluorescent staining of control (*w^1118^*/27.6) larval brains revealed the normal DA dorsolateral (Figure 5A) and medial (Figure 5B) patterns; as expected, there was no DTH immunofluorescence detected in the proventriculus (Figure 5C). However, in *elav*/27.6 larvae, DTH was expressed in essentially every cell in the CNS (Figure 5D), as well as in the 5-HT axonal proventricular fibers (Figure 5E). There is a 1:1 correspondence between the fibers revealed via 5-HT immunofluorescence and with anti-DTH (data not shown). Analysis of the progeny from *elav*Gal4 crossed with the 35.1 UAS transgene maintained over a larval marker, *Black cell *(*Bc^1^*) revealed the normal DA dorsolateral (Figure 5F) and medial (Figure 5G) patterns in *elav/Bc^1 ^*larval brains, but their *elav*/35.1 siblings displayed the same ubiquitous DTH immunofluorescence as did *elav*/27.6 animals (Figure 5H). This was quantitated by analyzing pixel intensity of the last pair of dorsolateral neurons in THGal4/35.1 and THGal4/27.6 animals (Figure 5I), since expression would then be limited to DA neurons. This analysis confirmed the increase in DTH levels in these transgenic animals.

Feeding behavior in animals with constitutive overexpression of neuronal DTH was reduced, similar to the DTH knockdowns (Figure 5J). To conclusively establish that this resulted from increased DA synthesis, 16-hour old control embryos (*w^1118^*;THA;THK) were exposed for the last 6 hours of embryogenesis either to serum-free media, or to serum-free media plus 10^-6 ^M DA-HCl, and assayed for feeding behavior as larvae. Only embryos exposed to exogenous DA during CNS development displayed a reduction in feeding (Figure 5J). As before, locomotor behavior of the larvae was normal (Figure 5K). Thus, levels of developmental neuronal DA above or below the normal level resulted in depressed feeding.

**Figure 6:**
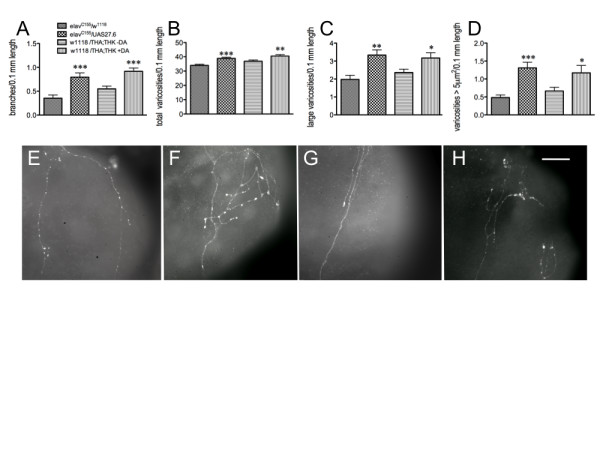
**Constitutive induction in neuronal DTH or increased systemic DA during late embryogenesis results in aberrant larval gut fiber architecture**. *elav^C155^*/UAS27.6, its parental control, *elav^C155^*/*w^1118^*, and 16 hr old control (*w^1118^;*THA;THK) embryos were dechorionated, devitillenized, and exposed to serum-free media with or without 10^-6^M DA for 6 hours until hatching. A. Branching. B. Varicosity number. C. Large varicosities. D. Varicosities > 5 μm^2 ^in diameter. *elav*/*w^1118^*, 28 fibers from 22 guts from 4 independent experiments; *elav^C155^*/27.6, 40 fibers from 32 guts from 7 independent experiments; *w^1118^*/THA;THK -DA, 45 fibers from 26 guts from 9 independent experiments; *w^1118^*/THA;THK + DA, 44 fibers from 34 guts from 13 independent experiments. *p < 0.05, **p < 0.01, ***p < 0.001, Student's unpaired t-test, comparing *elav*/*w^1118 ^*with *elav^C155^*/UAS27.6 and *w^1118^*/THA;THK -DA with *w^1118^*/THA;THK + DA. Lines above the graph depict standard error of the mean. E - H. Visual depiction of 5-HT axonal fibers from *elav*/*w^1118 ^*(**E**), *elav*/UAS27.6 (**F**), *w^1118^/*THA;THK -DA (**G**) and *w^1118^/*THA;THK + DA (**H**) guts. Scale bar = 30 μm.

Consistent with the perturbations in feeding, the 5-HT axonal fibers projecting to the gut from larvae with increased neuronal DA levels during development of the circuit display greater complexity relative to controls: increased fiber branching (Figure 6A), increased number of 5-HT-containing vesicles along the axon length (Figure 6B), and greater numbers of larger vesicles (Figure 6C, D). This can be observed visually when examining axonal fibers from *elav^C155^/w^1118 ^*(Figure 6E), *elav^C155^*/THA;THK (Figure 6F), *w^1118^*/THA;THK -DA (Figure 6G), and *w^1118^*/THA;THK + DA (Figure 6H). These data suggest that perturbations in developmental neuronal DA levels above or below a certain threshold can affect development of neural circuitry.

### Perturbations in neuronal DTH levels after larval CNS development do not affect feeding or fiber architecture

**Figure 7:**
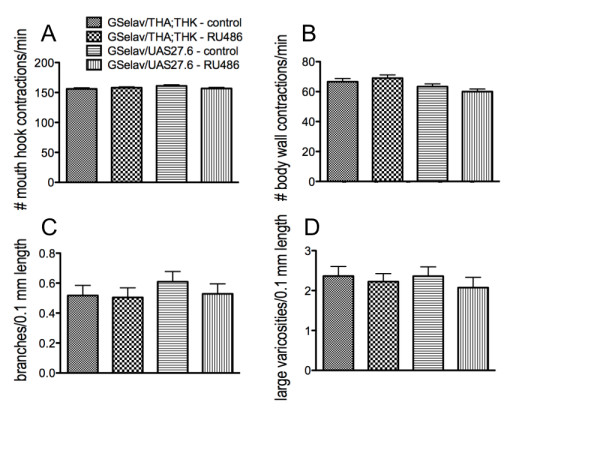
**Manipulation of neuronal DA levels after the larval CNS has developed has no effect on feeding behavior or gut fiber architecture**. Control - uninduced; RU486 - induced. A. Feeding. B. Locomotion. n = 40 for behavioral analyses, from 4-6 independent experiments. C. Number of branches per 0.1 mm length along the axonal fiber. D. Number of large varicosities per 0.1 mm length. GS*elav*/THA;THK uninduced control, 41 fibers from 34 guts from 7 independent experiments; GS*elav*/THA;THK induced (RU486), 41 fibers from 33 guts from 8 independent experiments; GS*elav*/THUAS27.6 uninduced control, 40 fibers from 30 guts from 6 independent experiments; GS*elav*/THUAS27.6 induced (RU486), 39 fibers from 28 guts from 9 independent experiments. Statistical analyses were performed using Student's t test comparing uninduced with induced animals for each genotype. Lines above the graph depict standard error of the mean.

Although previous studies demonstrated that systemic feeding of a TH inhibitor or of L-DOPA (the product of the TH reaction) to 2^nd ^instar larvae did not affect feeding behavior [31], we used an inducible *elav *driver to reduce (GS*elav*/THA;THK) or increase (GS*elav*/27.6) neuronal DTH expression in 2^nd ^instar larvae, after the larval nervous system had fully developed. As expected, there were no changes in feeding behavior (Figure 7A) or in locomotion (Figure 7B), and gut fiber architecture was also unchanged. Branching (Figure 7C), varicosity number (data not shown), varicosity area (data not shown) and number of large varicosities (Figure 7D) were unaffected by manipulation of neuronal DA levels after the nervous system had developed. Thus, whether DA levels are manipulated systemically in mature larvae, as opposed to during late embryogenesis, by pharmacological agents, or via targeted expression in the CNS, there is no effect on larval feeding. These results conclusively demonstrate that although DA neurotransmission does not modulate larval feeding behavior, neuronal DA is required during late embryogenesis for the normal development of the serotonergic feeding circuit.

**Figure 8:**
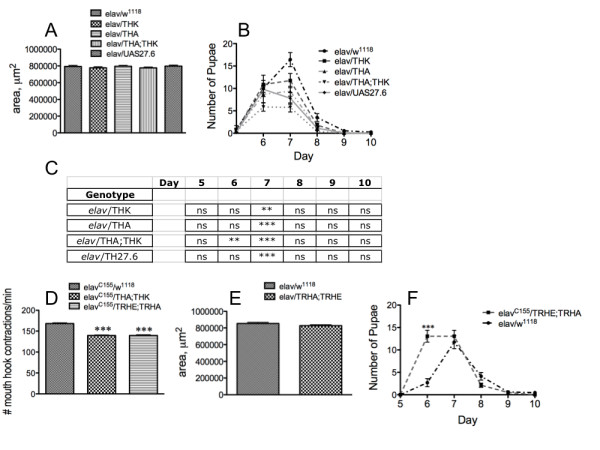
**Reduction in feeding does not affect organismal development**. 50 embryos from the genotypes *elav/w^1118 ^*(control), *elav*/THK, *elav*/THA and *elav*/THA;THK (increasing neuronal knockdown of DTH) and *elav*/UAS27.6 (increased DTH expression in the CNS), or *elav/w^1118 ^*(control) and *elav*/TRHE;TRHA, were established in 16 replicate vials. A - C. Manipulation of neuronal TH levels. A. Pupal size . B. Time to pupariation. C. Reduction in number of pupae. **p < 0.01, ***p < 0.001, two way ANOVA followed by Bonferroni's post-tests. D, comparison of feeding rate between *elav/w^1118^*, *elav*/THA;THK, and *elav*/TRHE;TRHA. ***p < 0.001, one way ANOVA followed by Dunnett's post-test. E - F. Manipulation of neuronal TRH levels. E. Pupal size for the DTRH double transgenic knockdown line. F. Increase in number of pupae. ***p < 0.001, two way ANOVA followed by Bonferroni's post-test.

Reduction in feeding does not affect time to pupariation or pupal size. *Drosophila *larvae must reach a critical size before pupariation can be initiated. Larvae with higher feeding rates are able to reach this critical size more quickly, and thus pupate faster [32,33]. We therefore assessed whether the reduced feeding rate resulting from impairment of the 5-HT feeding circuit as a consequence of reduced or increased embryonic DTH levels affected developmental or growth rates. 50 embryos from each genotype (*elav/w^1118^*, *elav*/THK, *elav*/THA, *elav*/THA;THK and *elav*/UAS27.6) were established in parallel in 16 independent vials containing standard food medium. At the same time each day the number of pupal cases in each vial was recorded, and pupae from each vial were removed and measured. There was no change in pupal size for animals with reduced or increased TH levels in the CNS (Figure 8A), and no change in time to pupariation (Figure 8B), but there was a direct correlation between the extent of neuronal TH knockdown or upregulated TH levels and the number of pupae (Figure 8C). The vials were observed over a period of several days, and all pupae that were viable were recorded; no pupae were ever observed after day 10. To determine whether the increase in lethality arose from reduced feeding, the same experiment was performed using animals with targeted knockdown of neuronal 5-HT synthesis (*elav/w^1118 ^*[control] and *elav*/TRHE;TRHA [a transgenic line containing two copies of the DTRH RNAi transgene]), since these animals display a similarly reduced feeding rate when compared with *elav*/THA;THK animals (Figure 8D). Again, there was no change in pupal size (Figure 8E) or time to pupariation (Figure 8F), but in this case, the reduced feeding rate in *elav^C155^*/TRHE;TRHA larvae correlated with *increased *pupal survival (Figure 8F). Therefore, changes in developmental rate are unlikely to be the consequence of reduction in feeding rate to 83%, the rate observed in both *elav^C155^*/THA;THK and *elav^C155^*/TRHE;TRHA animals.

### DA exerts its neurotrophic effects on the 5-HT circuit via the D2R receptor expressed in 5-HT neurons during embryonic CNS development

**Figure 9:**
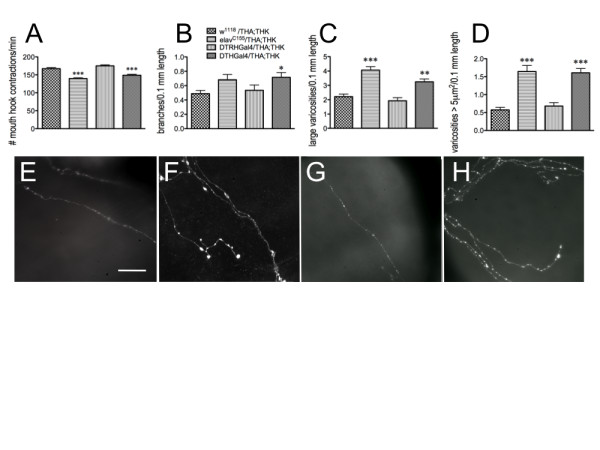
**The DA neurotrophic signal is released from DA neurons**. Knockdown of neuronal DTH synthesis in 5-HT neurons (DTRHGal4) and DA neurons (DTHGal4) was compared with pan-neuronal (*elav^C155^*) expression. A. Feeding. ***p < 0.001, one-way ANOVA followed by Tukey's multiple comparisons post-test. n = 40 animals for each genotype from 4-6 independent experiments. B - D. 5-HT gut fiber architecture. B. Branching. C. Number of large varicosities per 0.1 mm neurite length. D. Number of varicosities > 5 μm^2^. *p < 0.05, **p < 0.01, ***p < 0.001, one-way ANOVA followed by Tukey's multiple comparisons post-test. *w^1118^*/THA;THK, n = 66 fibers from 48 guts from 8 independent experiments; *elav*/THA;THK, n = 35 fibers from 18 guts from 3 independent experiments; DTRHGal4/THA;THK, n = 38 fibers from 24 guts from 10 independent experiments; DTHGal4/THA;THK, n = 31 fibers from 23 guts from 7 independent experiments. Lines above the graph depict standard error of the mean. E - H. Visual depiction of 5-HT axonal fibers from *w^1118^/*THA;THK (**E**), *elav^C155^/*THA;THK (**F**), DTRHGal4/THA;THK (**G**) and DTHGal4/THA;THK (**H**) guts. Scale bar = 30 μm.

To confirm that these developmental effects occurred via the actions of DA released from DA neurons, rather than any reduction in 5-HT via knockdown of DTRH, which shares some homology with DTH, the THA and THK transgenes were expressed in serotonergic neurons (using the DTRHGal4 driver) as well as in dopaminergic neurons (using the DTHGal4 driver) (Figure 9). As expected, reduction in feeding was observed only when the transgenes were expressed in DA neurons (Figure 9A). Similarly, the effects on gut fiber architecture occurred only when the transgenes were expressed in DA neurons. Increased branching (Figure 9B), increased numbers of 5-HT containing vesicles (Figure 9C), and increased numbers of large 5-HT-containing vesicles (Figure 9D) were observed only in fibers from *elav^C155^*/THA;THK and DTHGal4/THA;THK, and not in *w^1118^*/THA;THK and DTRHGal4/THA;THK, animals. This was observed visually when comparing *w^1118^*/THA;THK (Figure 9E), *elav^C155^*/THA;THK (Figure 9F), DTRHGal4/THA;THK (Figure 9G) and DTHGal4/THA;THK (Figure 9H) 5-HT axonal gut fibers.

**Figure 10:**
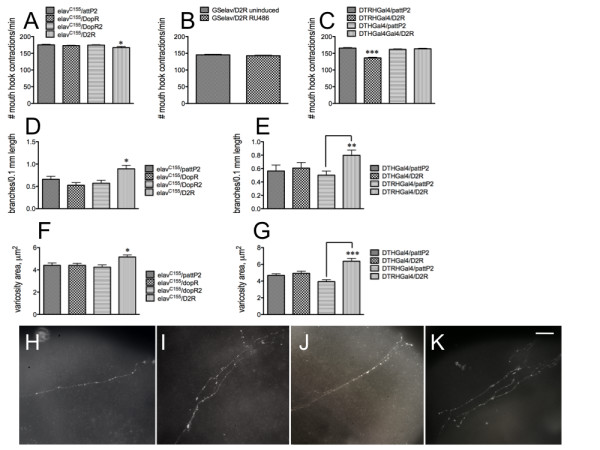
**DA exerts its effects via a D2R receptor expressed in 5-HT neurons during development of the circuit**. A - C. Feeding behavior. A, Only the D_2 _receptor, D2R, affects feeding behavior (*elav^C155^*/pattP2, parental control; DopR and DopR2, D_1 _receptors). B. Neuronal knockdown of D2R expression in 2^nd ^instar larvae has no effect. C. D2R expression must be reduced in 5-HT (DTRHGal4), but not DA (DTHGal4), neurons, to affect feeding behavior. n = 40 for behavioral analyses, from 4-6 independent experiments. *p < 0.05, ***p < 0.001. Statistical analyses were performed using one way ANOVA and Dunnet's post-test (**A**, **C**) or Student's t-test (**B**). D - G. Axonal fiber architecture. D - E. branching. F - G. Varicosity area. *p < 0.05, **p < 0.01, ***p < 0.001. Statistical analyses were performed using one way ANOVA and Dunnet's post-test (**D**, **F**) or Student's t-test (**E**, **G**). *elav^C155^*/pattP2, 43 fibers from 32 guts from 7 independent experiments; *elav^C155^*/DopR, 47 fibers from 37 guts from 6 independent experiments; *elav^C155^*/DopR2, 37 fibers from 29 guts from 5 independent experiments; *elav^C155^*/D2R, 37 fibers from 22 guts from 4 independent experiments; DTHGal4/pattP2, 34 fibers from 26 guts from 3 independent experiments; DTHGal4/D2R, 29 fibers from 18 guts from 5 independent experiments; DTRHGal4/pattP2, 33 fibers from 26 guts from 5 independent experiments; DTRHGal4/D2R, 27 fibers from 22 guts from 3 independent experiments. Lines above the graph depict standard error of the mean. H - K. Visual depiction of 5-HT axonal fibers from *elav^C155^/*pattP2 (**H**), *elav^C155^/*D2R (**I**), DTRHGal4/pattP2 (**J**) and DTRHGal4/D2R (**K**) guts. Scale bar = 20 μm.

Since DA must signal through a DA receptor, we used the *elav^C155 ^*driver to express dsRNA transgenic constructs for the two *Drosophila *D_1 _DA receptors (DopR and DopR2) as well as the single *Drosophila *D_2 _receptor, D2R. Only reduction in D2R expression had any effect on feeding (Figure 10A) when compared with the *elav^C155^*/pattP2 parental control. To confirm that D2R function was required during development of the circuit, we used the inducible *elav *driver GS*elav *in 2^nd ^instar larvae to reduce D2R expression, and as expected, saw no effect on feeding (Figure 10B). More critically, when using the DTRHGAL4 and DTHGal4 drivers to reduce expression of the D2R receptor in serotonergic or dopaminergic neurons, respectively, the effect on feeding was only observed when D2R expression was reduced in 5-HT neurons (Figure 10C), suggesting that dopamine exerts its effects on the 5-HT feeding circuit via signaling through the D2R receptor expressed in 5-HT neurons during development of the circuit. Again, the changes in feeding correlated with changes in the proventricular 5-HT axonal fiber architecture. Reduced DA signaling resulted in increased branching (Figure 10D, E) and varicosity area (Figure 10F, G), due to increased numbers of larger (greater than 5 μm^2 ^in diameter) 5-HT-containing vesicles (compare *elav^C155^*/pattP2, Figure 10H with *elav^C155^*/D2R, Figure 10I, and RH/pattP2, Figure 10J with DTRH/D2R, Figure 10K).

### Disparate relative levels of neuronal DA and 5-HT during development of the feeding circuit affect the function and architecture of the mature 5-HT feeding circuit

**Figure 11:**
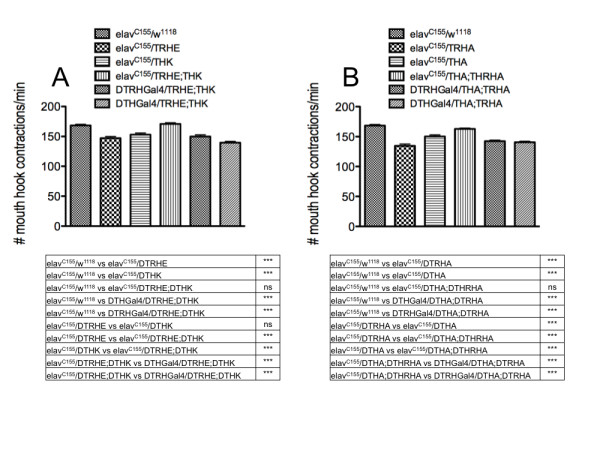
**Neuronal knockdown of both TRH and TH during development of the circuit results in normal feeding**. *elav^C155^*, pan-neuronal driver; DTRHGal4, drives expression in 5-HT neurons; DTHGal4, drives expression in DA neurons. A. TRHE, THK, weaker RNAi transgenes. B. TRHA, THA, stronger RNAi transgenes. n = 40 for behavioral analyses, from 4-6 independent experiments. ***p < 0.001, one way ANOVA followed by Tukey's multiple comparisons post-test. Lines above the graph depict standard error of the mean.

To assess whether neuronal DA and 5-HT interact with each other for normal development of the feeding circuit, we generated lines with different combinations of the DTH and DTRH transgenic RNAi constructs, and placed them under the control of the *elav^C155^*, DTHGal4 and DTRHGal4 drivers (Figure 11). Both THK and TRHE, when independently driven by *elav*, effect a reduction in larval feeding; however, there is no reduction in feeding in *elav*/TRHE;THK larvae (Figure 11A). When the same transgenic combination is placed under the control of either the DTHGal4 or DTRHGal4 driver, feeding is reduced, consistent with knockdown of only DA or 5-HT synthesis, but not both. Similar results were observed using the THA;TRHA transgenic combination, which results in stronger knockdown of TH and TRH (Figure 11B). These results suggest that when *both *neuronal DA and 5-HT levels are reduced, there is no net effect on feeding; feeding rate is perturbed only when DA levels are reduced, and normal 5-HT levels are maintained, or vice versa.

**Figure 12:**
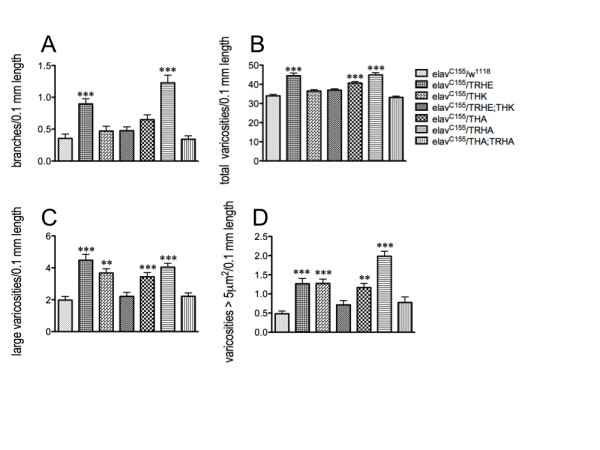
**Neuronal knockdown of both DTRH and DTH during CNS development results in normal gut fiber architecture**. *elav^C155^*, pan-neuronal driver; TRHE, THK, weaker RNAi transgenes; TRHA, THA, stronger RNAi transgenes. A. Branching. B. Total varicosity number per 0.1 mm length. C. Number of large (> 1 μm^2^) vesicles per 0.1 mm length; D. Varicosities > 5 μm^2 ^per 0.1 mm length. *elav^C155^*/*w^1118^*, 28 fibers from 22 guts from 4 independent experiments; *elav^C155^*/TRHE, n = 48 projecting fibers from 34 independent gut dissections; *elav^C155^*/THK, n = 40 fibers from 18 guts from 2 independent experiments; *elav^C155^*/TRHE;THK, n = 39 fibers from 28 guts from 6 independent experiments; *elav^C155^*/THA, n = 37 fibers from 21 guts from 2 independent experiments; *elav^C155^*/TRHA, n = 58 projecting fibers from 39 independent gut dissections; *elav^C155^*/THA;TRHA, n = 33 fibers from 18 guts from a single experiment. **p < 0.01, ***p < 0.001. Statistical analyses were performed using one way ANOVA and Dunnet's post-tests. Lines above the graph depict standard error of the mean.

These effects were also observed for the gut fiber architecture: when either neuronal DA or 5-HT synthesis was reduced, depending upon the strength of the knockdown, the fibers displayed increased branching, which was rescued to control levels in the presence of both transgenes (Figure 12A). Total varicosity number along the neurite length (Figure 12B), number of large vesicles (Figure 12C) and number of vesicles exceeding 5 μm^2 ^in diameter (Figure 12D) were also increased when *either *DTH or DTRH levels were reduced. However, when *both *DTH and DTRH levels were reduced, the gut fiber architecture was indistinguishable from that of controls.

Increased neuronal 5-HT during development of the circuit results in increased larval feeding and reduced complexity of the 5-HT axonal projections to the gut [17], and increased developmental DA has the opposite effect (Figures 5, 6). One would thus expect that increasing levels of both biogenic amines would result in normal feeding and gut fiber appearance, and this is what is observed (data not shown). This is also true for *elav*/THA;THK embryos exposed to exogenous 5-HT during the last 6 hours of embryogenesis (data not shown). These data provide evidence for interactions between DA and 5-HT during development of the 5-HT feeding circuit, perhaps to serve as inhibitory "checks" on their respective signaling pathways to generate a functional stomatogastric circuit.

## Discussion

The function of mature neural networks is absolutely dependent upon their development, which requires a complex interplay of numerous morphogenetic events including neurogenesis, migration, neurite outgrowth and synaptogenesis.

Disturbances in the development of neural networks likely underlie the etiology of neuropsychiatric diseases such as schizophrenia, and pervasive developmental disorders such as autism. It is therefore critical to identify factors necessary for normal CNS development, since they may serve as biomarkers for pathological disease states as well as possible avenues for therapeutic intervention. To facilitate elucidation of these factors, we have characterized a simple neural circuit, the 5-HT stomatogastric feeding circuit in the *Drosophila *larva. We demonstrated an inverse relationship between neuronal 5-HT levels during development of the circuit and the complexity of the 5-HT axonal fibers projecting from the larval brain to the foregut, which correlate with perturbations in feeding, the functional output of the circuit. In mammals, the neurotransmitter actions of DA modulate the motivation or anticipation of feeding behavior, as opposed to the behavior itself [e.g., [34-36].] Rats in which mature DA neurons have been lesioned with 6-hydroxydopamine display severe aphagia, but can recover from this treatment to eat normally [37]. Our data show that induction or reduction of neuronal DA synthesis in the larval stage has no effect on feeding. However, perturbations in DA levels during late embryogenesis have a significant effect on the function of the 5-HT feeding circuit.

The homology between DTH and its sister enzyme DTRH at the DNA level is sufficiently limited that neuronal expression of the DTH RNAi and UAS constructs should not affect DTRH expression [30,38], Figure 9]. While DTRH protein was ectopically observed in the CNS when DTRH expression was induced using a UAS transgene, the 5-HT pattern was unchanged except for intensity, implying that all the necessary factors for 5-HT synthesis (substrate, cofactor) were only available in 5-HT neurons [17]. The analogous experiment for DA could not be assessed in *elav^C155^*/UASDTH animals due to the lack of an anti-dopamine antibody competent for our immunohistochemical studies. However, it is reasonable to assume that DA synthesis is also not induced in non-DA neurons, as these cells would lack dopa decarboxylase, the second enzyme in DA synthesis, as well as the cofactor tetrahydrobiopterin, and sufficient amounts of the substrate tyrosine. Since DTH also forms a complex with GTP-cyclohydrolase, the rate-limiting enzyme in tetrahydrobopterin synthesis, and this complex is necessary for DTH activity [39], it is likely that while DTH protein may be expressed in other neurons, its activity would be significantly compromised. This is consistent with the observation that late-stage embryos exposed to exogenous DA recapitulate the feeding and gut fiber phenotypes observed with *elav^C155^*/UAS DTH animals, since the DA transporter is only expressed in DA neurons [40]. Thus, our approach permitted controlled and specific manipulation of neuronal DA synthesis.

### Dopamine plays a critical role in development of the 5-HT feeding circuit although it does not act as a transmitter to modulate feeding behavior

Neuronal expression of two independent DTH RNAi transgenes singly as well as in combination demonstrated that the level of DTH knockdown could be titrated; this correlated with increased complexity of the 5-HT axonal gut fibers as well as with deficits in feeding rate. When these transgenes were expressed after the circuit was mature, in 2^nd ^instar larvae, there were no effects observed on either feeding or gut fiber appearance. More importantly, the defects resulting from constitutive knockdown of DTH expression were fully rescued by exposure to DA during the last 6 hours of embryogenesis, when the 5-HT feeding circuit is developing, confirming a role for DA in the circuit's normal development and mature function. It can be argued that high levels of exogenous DA could be taken up by the serotonin transporter and metabolized via monoamine oxidase activity, resulting in toxicity of the 5-HT neurons. However, *Drosophila *do not catabolize DA via this route since they lack these enzymes [41]. Excess dopamine is modified and cross-linked into the chitinous exoskeleton, and thus the cuticle is the dopamine "sink." Therefore, not only is it unlikely that sufficient amounts of DA would be taken up by dSERT into 5-HT neurons, DA would not be degraded into a toxic metabolite. Additionally, since reduction in developmental 5-HT levels increases complexity of the fibers [17], one would expect, if DA was toxic to 5-HT neurons, that the complexity would be further increased, rather than reduced to normal levels. Constitutive overexpression of DTH, or exposure of wild-type embryos to exogenous DA during the last six hours of embryogenesis, also resulted in reduced feeding and more complex gut fiber architecture. Lastly, our observation that the actions of DA on the feeding circuit occur via a D_2_ receptor expressed in 5-HT neurons results in enhanced complexity rather than reduced complexity of the fibers, also argues against this mechanism. Thus, unlike 5-HT, the development of the circuit is sensitive to a threshold for DA levels, above or below which affects developmental signaling pathways. A similar response for DA has been observed in mammals [42], as well as in zebrafish [reviewed in [43]], and may reflect conserved signaling mechanisms across species.

In mammals, neonatal depletion of DA results in an array of behavioral perturbations in relatively simple motor tasks such as locomotion [44], as well as in higher order cognitive function [45]. It has also been shown to affect cortical morphogenesis [46], implying a direct correlation between changes in neural circuitry caused by altered neuronal DA levels during a vulnerable and plastic developmental period, and the behavioral deficits observed in the animal. Depletion of cortical DA in neonatal mice results in changes in gene expression in factors necessary for axon guidance and dendritic growth, as well as those required for folding actin and tubulin [47]. Lesioning neonatal DA neurons via 6-hydroxydopamine is also known to induce sprouting of 5-HT axonal fibers in the rat cerebral cortex [48]. Our results suggest that the morphogenic role for DA in neural circuit development, and its interactions with 5-HT signaling pathways, may be an evolutionarily conserved mechanism.

### Neuronal DA and 5-HT affect pupal development, but not as a consequence of reduced feeding

In order to leave the food medium in preparation for pupariation, *Drosophila melanogaster *larvae must reach a minimum mass; in general, this occurs early in the 3^rd ^larval instar [49]. Slower feeding larvae take longer to reach this critical weight [32]. When neuronal DTH levels are either decreased or increased during late embryogenesis, larval feeding rate is reduced, but while there is an increase in lethality, neither pupal size nor time to pupal formation is affected (Figure 8). This cannot be attributed to a reduced feeding rate, however, since animals carrying two copies of the DTRH RNAi transgene display a more robust survival rate than controls; pupal size and time to pupation are the same. The feeding rate is depressed to approximately 85% of normal in the both *elav*/THA;THK and *elav*/TRHE;TRHA animals. Thus, while both neuronal DA and 5-HT modify the larval feeding circuit and thus its functional output in the larva, the survival outcomes must arise as a consequence of perturbations of other neural circuits. A role for DTRH in the developing brain has long been established [50], and DA has been shown to play a critical role in development in *Drosophila *[31,51]. Therefore, a reduction or increase in neuronal DA synthesis, or a reduction in neuronal 5-HT synthesis, in late embryogenesis affects the development and function of the feeding circuit, but alterations in this circuit arising from these perturbations have no direct effect on general growth parameters.

### DA exerts its neurotrophic effects on the 5-HT circuit via the D2R receptor expressed in 5-HT neurons during embryonic CNS development

An *in vitro *role for D2R in neuronal development has been established, since it was identified in a screen for RNAi phenotypes that altered growth of primary neural cells in culture; in these studies, reduced D2R expression resulted in increased neurite branching [52]. Our results demonstrate an *in vivo *developmental role for D2R in CNS development. In mice, blockade of the D_2 _receptor during striatal development has been shown to result in increased sprouting of axonal fibers [53]. The D_2 _receptor in larval zebrafish transiently regulates a swim circuit, which may be critical for the development of the mature circuit [54]; it also modulates levels of the protein kinase Akt, resulting in perturbations in development of GABAergic neuronal pathways [43]. Thus, our results are consistent with vertebrate studies demonstrating a key role for the D_2 _receptor in neural circuit development. There are three D_2_-like receptors in mammals: D_2_, D_3 _and D_4_, but while there is only a single gene encoding a D_2 _receptor in *Drosophila*, there are 8 protein isoforms that arise as a consequence of alternative splicing, with differences in the length and sequence of the third intracellular loop [55]. While D2R is expressed at high levels during the latter stages of embryogenesis, the spatial localization has been determined only in the larval and adult CNS [56], and it is unknown which isoform(s) mediate the developmental effects of DA on the 5-HT feeding circuit.

Both dopamine and serotonin have been shown to promote as well as inhibit axonal outgrowth [13,14,57-59]. Released 5-HT can induce growth cone collapse, resulting in reduced neurite branching in molluscan cerebral giant cells, acting via 5-HT receptors located in the growth cone as well as along the connective [reviewed in [59]. Since the altered axonal fibers in our study arise from 5-HT neurons, and since DA exerts its effect via a D_2 _receptor expressed in serotonergic neurons, dopamine may directly regulate the extent of neurite outgrowth from the central 5-HT neurons in the stomatogastric circuit. However, classic studies in *Lymnaea *have demonstrated that DA can act as a diffusible substance to promote neurite outgrowth and synapse formation [reviewed in [60,61]. Our studies do not exclude this possibility in *Drosophila*, but suggest that mechanisms are in place for the DA signals arising from DA neurons to both directly and indirectly affect the sprouting of 5-HT axonal fibers from specific central 5-HT neurons.

### Neuronal DA and 5-HT interact during CNS development to generate the mature feeding circuit

6-hydroxydopamine lesions in neonatal rats affect 5-HT axonal sprouting in the striatum, which does not occur in similarly lesioned adults [48,62,63]. While there is significant loss of DA neurons after 6-hydroxydopamine lesioning, which is not observed in the *Drosophila *transgenic lines in which tyrosine hydroxylase has been specifically reduced within central DA neurons, the net effect in both systems is a loss of DA release. Therefore, the neurotropic effects of DA on 5-HT axonal fibers that we have observed in our studies are consistent with mammalian studies, and again suggest that the developmental interactions between DA and 5-HT are likely to be evolutionarily conserved.

Our results differ from those of Budnik et al. [25] who examined the 5-HT stomatogastric feeding circuit in *Drosophila *larvae unable to synthesize dopa decarboxylase (DDC), the second step in the biosynthetic pathway for both dopamine and serotonin. In this study, they found that the fibers projecting from the proventriculus into the midgut displayed greater branching and increased numbers of varicosities; in our study, which assessed the fibers as they entered the proventricular foregut, we found that reduction of both neuronal DA and 5-HT synthesis during development of the circuit had no effect on the axonal fiber architecture, consistent with the normal feeding behavior observed in these animals. It is possible that the developing axonal projections could be exposed to different morphogenic environments as the neurite length extended during development from the foregut to the midgut. Since Budnik and colleagues eliminated DDC in every tissue, while our transgenics (*elav*/TRHE;THK and *elav*/THA;TRHA) specifically reduced central DA and 5-HT synthesis, the two studies may not be directly comparable.

How do dopamine and serotonin interact to effect changes in axonal arborization and vesicle populations? A number of studies have shown that neurotransmitters, including dopamine and serotonin, regulate dendritic transport along microtubules [reviewed in [64], and their activities in interacting with factors that stabilize or destabilize microtubules would also affect axonal stability. Dysfunctional microtubules result in aberrant neurotransmission. It is possible that that dopamine and serotonin may interact directly with different factors necessary for microtubule stabilization, via expression and/or post-translational modifications of microtubule-associated proteins. Changes in stabilization of the microtubule would then affect axonal outgrowth and branch development. These changes in the microtubule network would result in aberrant signaling of the 5-HT stomatogastric circuit, with perturbed feeding as the functional outcome. Past studies have demonstrated a direct role for DA in altering the phosphorylation state of MAP2, which is critical for dendritic maturation [65]. Alternatively, DA-receptor-mediated signaling might influence expression of neurotrophins by transactivation of other G protein coupled receptors. Activation of the D_1 _receptor in cultured rodent embryonic striatal neurons results in increased TrkB cell surface expression; TrkB is a high-affinity receptor for several neurotrophic factors including brain-derived-neurotrophic factor [66].

The DA neurons first appear 1-2 hours after the 5-HT neurons, suggesting there may be temporal constraints in the developmental pathway - that is, DA may exert its influence during a limited window of the time required for development of the feeding circuit. The identification of the factors by which dopamine and serotonin affect axonal maturation in this circuit is currently under investigation.

## Conclusions

These studies demonstrate a developmental role for dopamine on a neural circuit, even though it does not act as a transmitter to modulate the functional behavioral output of this circuit. This work also shows that dopamine and serotonin interact with each other to generate the neural architecture necessary for the circuit's normal function.

## Methods

### Fly culture

Flies were maintained in glass pint bottles containing standard agar-cornmeal-yeast food at 25°C on a 12 hour light-dark cycle. Staged larvae for behavioral studies were collected from population cages maintained at 25°C on a 12 hour light-dark cycle. Females were allowed to lay eggs overnight on apple juice-agar plates, and newly hatched larvae were collected by maintaining plates with newly deposited eggs at 25°C for 24 hr, and collecting 1^st ^instars by migration of the animals onto yeast paste in the center of the agar plate. Early 3^rd ^instar larvae were obtained by allowing 1^st ^instars to age for 48 hr.

### Fly Strains

All stocks were obtained from the Bloomington, Indiana stock center unless otherwise noted. *w^1118 ^*is the parental strain for the *Drosophila *tyrosine hydroxylase (DTH) and *Drosophila *tryptophan hydroxylase (DTRH) UAS and RNAi transgenic lines. These flies are isogenic for the second and third chromosomes. DTH and DTRH are the first and rate-limiting enzymes in the synthesis of DA and neuronal 5-HT, respectively [30,38]. Two independent DTRH RNAi transgenic lines were used: TRHE, on chromosome 2, and TRHA, on chromosome 3 [17]; these were combined (*w^1118^*; TRHE;TRHA) to increase knockdown of DTRH expression levels, and thus, 5-HT synthesis.*y^1 ^v^1^; P{y^+t7.7^v^+t1.8^= TRiP. HM04077}attP2 *expresses a dsRNA for the D_1 _dopamine receptor DopR under UAS control; *y^1^v^1^; P{y^+t7.7^v^+t1.8^= TRiP. JF02043}attP2 *expresses a dsRNA for the D_1 _dopamine receptor DopR2 under UAS control; and *y^1^v^1^; P{y^+t7.7^v^+t1.8^= TRiP.JF02025}attP2 *expresses a dsRNA for the D_2 _dopamine receptor D2R under UAS control.

*pP{w^+mW.hs^= GawB}elav^C155 ^*is a pan-neuronal Gal4 driver and constitutively drives expression in post-mitotic neural tissue; it initiates expression beginning after embryonic stage 9, peaking within the next few hours, and decreasing afterwards [67]. *pP{ELAV-GeneSwitch} *is an inducible pan-neuronal Gal4 driver referred to as GS*elav *(the kind gift of Haig Keshishian (Yale University, New Haven CT). DTRHGal4 drives expression in serotonergic neurons [68], and was the gift of Dr. Ed Kravitz (Harvard University, Cambridge MA). The DTHGal4 line drives expression in the majority of dopaminergic neurons as well as in other tissues where tyrosine hydroxylase is expressed (generated by Serge Birman, Developmental Biology Institute of Marseille, Marseille, France). The P {w^+mW.hs^= GawB}elav^C155^, P{^w+mC^= UAS-syt.eGFP}1, w*, was used to label synaptic vesicles using green fluorescent protein.

### Generation of transgenics

Complementary DNA encoding full-length DTH was subcloned into both the SympUAS vector [69] downstream of the yeast Gal4 UAS (to generate DTH dsRNA) and a standard pUAS vector (stock no. 1000, *Drosophila *Genomics Resource Center). These constructs were injected into *w^1118 ^*embryos using standard transformation techniques [70], and were generated using the services of Genetic Services Inc. (Cambridge MA).

### Induction of expression using the GS*elav *driver

The GSelav construct contains a human progesterone receptor-ligand-binding domain, which binds to Upstream Activator Sequences (UAS) in the presence of the antiprogestin RU486 (mifeprestone, Sigma, St. Louis MO) to induce expression of the UAS fused to the transgene of interest. 2^nd ^instar lavae were submerged in either RU486 (6 mg/ml in 80% ethanol, induced) or 80% ethanol (uninduced control) for two minutes, and aged for 24 hr (quantitation of neuronal DTH levels) or for 40-44 hr (reduction in DA levels for behavioral analyses and immunohistochemistry of the 5-HT gut fibers). Uninduced animals serve as controls, since expression of the transgene occurs only in animals exposed to the ligand. The DTH sequences used are unique and will not affect expression of any other gene.

### Analysis of DTH expression in the transgenic lines

Changes in fluorescent intensity of specific neurons were visualized with an antibody raised against DTH [71] as described in [17]. The DA cell pattern in each brain was visualized under fluorescence after incubation of dissected larval brains with anti-DTH, and photographed at the same magnification and exposure. Relative DTH levels were assessed by dissecting larval brains from each genotype (*elav^C155^/w^1118^, elav^C155^*/THK, *elav^C155^*/THA, and *elav^C155^*/THA;THK or THGal4/*w^1118^*, THGal4/UAS35.1 and THGal4/UAS27.6). Each fly carried either one copy of the Gal4 driver and one copy of the RNAi or UAS transgene, or the driver in combination with *w^1118^*, the parental line for the generation of all transgenic lines, which served as a control. Brains were dissected from each genotype and assessed in parallel under identical conditions. The average density of pixel intensity was sampled from seven regions within each neuron from each brain from each genotype, covering the entire cytoplasmic region (Northern Eclipse, Empix Imaging, North Tonawanda, NY, USA). *elav/w^1118^*, n = 30 neurons [control]; *elav*/THK, n = 18 neurons; *elav*/THA, n = 15 neurons; *elav*/THA;THK, n = 13 neurons; THGal4/*w^1118^*, n = 30 neurons [control]; THGal4/THK, n = 22 neurons; THGal4/THA, n = 18 neurons; THGal4/THA;THK, n = 26 neurons; THGal4/*w^1118^*, n = 12 neurons [control]; THGal4/UAS35.1, n = 24 neurons; THGal4/UAS27.6, n = 15 neurons. Statistical analyses were performed using one-way ANOVA followed by Tukey's post-test.

### Immunohistochemistry of proventricular tissue

The proventriculus and the midgut from wandering 3^rd ^instar larvae were dissected in phosphate-buffered saline (PBS), fixed for 1 hour (4% EM-grade formaldehyde in 1× PBS) and washed thoroughly in PBT (1× PBS, 0.1% protease-free bovine serum albumin, 0.1% Triton-X-100). Gut tissues were dissected from wandering 3^rd ^instars to allow for clearance of the yeast (food) from the gut; incomplete clearance interfered with immunohistochemical analyses. Tissues were incubated in primary antibody, washed in PBT, and incubated in secondary antibody (Alexa Fluor 568 goat anti-mouse or anti-rabbit IgG; 1:400 dilution; Invitrogen - Molecular Probes, Carlsbad, CA, USA). Tissues were then incubated in 4 mM sodium carbonate, mounted in 4% n-propyl gallate in 20 mM sodium carbonate, and viewed under fluorescence. Primary antibodies used included anti-5-HT (Spring Biosciences, CA, USA), anti-DTH [71], and anti-DTRH [30]. To enhance 5-HT immunoreactivity, dissected gut tissues were pre-incubated in 10^-6^M 5-HT for one hour at room temperature before extensive washing and incubation with the primary antibody; this concentration of 5-HT after tissue fixation does not affect neuronal architecture or varicosity density in immunohistochemical analyses [17].

### Analysis of neuronal circuitry

Serotonergic fibers projecting to the proventriculus from each genotype were assessed immunohistochemically and photographed at the same resolution. Quantification of varicosities and branching of fibers were analyzed using Neuroleucida, version 5 and Neuroexplorer (MBF Bioscience, Chicago, IL, USA). Individual projections were traced within the body of the proventriculus, and varicosity number, branches, and the number of large varicosities per 100 μm length was quantified. Area (μm^2^) per large varicosity was also determined, as was the number of varicosities 5 μm^2 ^or greater in diameter along the neurite length. A varicosity was defined as a bright, discrete unit sufficiently enlarged beyond the size of the fiber, and large varicosities were defined as those greater than 1 μm^2 ^(i.e., larger than the width of the neurite fiber). In general, between 25 and 40 individual fibers were examined from over 20 animals in at least 4 independent experiments.

### Behavioral paradigms

#### Feeding

A single late 2^nd ^- early 3^rd ^instar larva was placed in the center of a 2% agar-filled petri dish overlaid with a 2% yeast solution, and the number of mouth hook contractions was counted for one minute after a 30 s acclimation period [31]. The animals were matched for age, and assessed for feeding at this stage, since feeding rate is relatively constant at that age [29]. The rate of mouth hook contractions directly correlates with the amount of food ingested [72]. n = 40 for each genotype. Feeding rate distributions for each genotype follow a Gaussian distribution.

### Locomotion

Each animal was placed on a 2% agar substrate and allowed to acclimate for 30 seconds. The larva was then observed as it crawled over the substrate for a period of one minute, and each posterior to anterior contractile wave was counted. The contractile motions are initiated by the extension and retraction of the larval mouth hooks in the agar surface [31]. n = 40 for each genotype. In general, the same animal was first assessed for locomotor behavior, and then for feeding.

### Statistics

Statistical analyses were accomplished by one-way ANOVA using Tukey's or Dunnett's post-hoc tests (for constitutive knockdown with *elav^C155 ^*Gal4) or by Student's t-test (for temporal knockdown with GS*elav *or for embryonic studies). n = 40 animals from 4-6 independent experiments.

### Embryonic exposure to 5-HT or DA

Staged embryos were aged until 16 hr after egg laying, dechorionated and devitellinized by exposure to octane (Sigma Aldrich, electronic grade) [17]. Embryos were incubated in 10^-6 ^M 5-HT or 10^-6 ^M DA, or both, in serum-free medium (Sf-900 II SFM 1X, GIBCO) for 4-6 hours until hatching, and then placed in yeast paste on an agar plate and kept at room temperature.

### Developmental analyses

50 embryos from each genotype (*elav/w^1118^*, *elav*/THK, *elav*/THA, *elav*/THA;THK and *elav*/UAS27.6, or *elav/w^1118 ^*and *elav*/TRHE;TRHA) were established in parallel in 16 independent vials. At the same time each day the number of pupal cases in each vial was recorded. In addition, pupae from each vial were removed and measured. The area of each pupa was quantified by photographing them at the same exposure and magnification (n = 50), and drawing the perimeter around each using Neuroexplorer (MBF Bioscience, Chicago, IL, USA). Statistical analyses for developmental timepoints were accomplished using two way ANOVAs followed by Bonferroni post-tests, and for pupal size by one-way ANOVAs followed by Dunnett's post-tests.

## Authors' contributions

WSN designed the experiments, quantitated *in vivo *transgene expression and performed the developmental analyses, participated in the behavioral and immunohistochemical experiments and the statistical analysis, and drafted the manuscript. PB participated in the behavioral and immunohistochemical experiments and the statistical analysis. Both authors read and approved the final manuscript.
